# Clinicopathologic characterization of intradiverticular carcinoma of urinary bladder - a study of 22 cases from a single cancer center

**DOI:** 10.1186/s13000-014-0222-8

**Published:** 2014-11-26

**Authors:** Hua Zhong, Saby George, Eric Kauffman, Khurshid Guru, Gissou Azabdaftari, Bo Xu

**Affiliations:** Department of Pathology and Laboratory Medicine, Roswell Park Cancer Institute, Elm and Carlton Streets, Buffalo, NY 14263 USA; Department of Medicine, Roswell Park Cancer Institute, Elm and Carlton Streets, Buffalo, NY 14263 USA; Department of Urology, Roswell Park Cancer Institute, Elm and Carlton Streets, Buffalo, NY 14263 USA; Current address: Department of Pathology and Laboratory Medicine, Robert Wood Johnson Medical School, UMDNJ, One Robert Wood Johnson Place - MEB 212, New Brunswick, NJ 08901 USA

**Keywords:** Urinary bladder, Intradiverticular carcinomas, Histopathologic features, Clinical outcome

## Abstract

**Background:**

To examine histopathologic features and clinical outcomes of intradiverticular bladder carcinomas.

**Methods:**

Twenty-two consecutive patients with intradiverticular bladder carcinoma treated with either endoscopic transurethral resection or partial or radical cystectomy at a single institution between years of 1995 to 2011. Clinicopathologic characteristics and oncologic outcomes of patients were retrospectively analyzed, including tissue histology re-review by genitourinary pathologists.

**Results:**

Histologically, 9 cases (41%) were non-invasive papillary urothelial carcinoma, 13 cases (59%) were invasive urothelial carcinoma, including three cases of small cell carcinoma. Final pathology revealed synchronous extradiverticular urothelial carcinomas in 6 out of 9 cases (67%) of non-invasive and 2 out of 10 cases (20%) invasive intradiverticular urothelial carcinoma, respectively. More than half of cases (13/22, 59%) showed a distinctive hypertrophic layer of muscularis mucosae. There was no statistical difference in disease free survival or overall survival between non-invasive and invasive tumors within approximately 3 years of follow up (mean 38 months, median 32 months). While stage T3 patients generally did poorly, oncologic outcomes for stage T1 patients were no different than those of stage Ta.

**Conclusion:**

Intradiverticular carcinomas are often associated with a hypertrophic layer of muscularis mucosae that can potentially confound tumor staging. Non-invasive intradiverticular urothelial carcinomas are more likely to have coexisting synchronous extradiverticular lesions. The absence of a muscularis propria layer may not necessarily predispose T1 tumors to more aggressive disease.

**Virtual Slides:**

The virtual slide(s) for this article can be found here: http://www.diagnosticpathology.diagnomx.eu/vs/13000_2014_222

## Background

Intradiverticular bladder carcinoma is defined as a malignant epithelial neoplasm arising within a diverticulum of urinary bladder. The most common intradiverticular malignancy is urothelial carcinoma. Bladder diverticula are caused by congenital or acquired defects which cause an outpouching of bladder wall and it usually lacks the muscularis propria layer (detrusor muscle) [1]. Most bladder diverticula are small and asymptomatic. A subset of these lesions, however, may be complicated with inflammation, calculus, infection and malignancy [2,3].

Compared to that of anatomically normal urinary bladder wall, intradiverticular urothelial lining is known to have higher carcinoma rate with intradiverticular bladder carcinoma occurring in approximately 10-14% and up to 50% of all bladder diverticula [2-5]. However, only 1.3% of urothelial carcinomas arise from vesical diverticula in 611 patients treated for bladder tumor [6]. Due to its rarity, intradiverticular carcinoma remains infrequently encountered in general practice. As result, the histopathologic features and clinical outcomes of intradiverticular bladder carcinoma are not well investigated, with only three studies of notable size in the current literature, each including less than 40 patients [2,3,7,8]. While absence of muscularis propria layer may in theory predispose intradiverticular tumors to a greater risk for bladder wall infiltration and possibly spreading into adjacent organs, the aggressiveness of these tumors and their clinical outcomes remain poorly characterized. In this study, we aimed to expand current understanding of this unique neoplasm presentation by characterizing the histopathologic features and clinical outcomes of 22 patients with intradiverticular bladder carcinoma.

## Methods

### Case selection

This study was approved by the Roswell Park Institutional Review Board.

Twenty-two consecutive cases of intradiverticular carcinoma were identified from institutional surgical pathology archive spanning from 08/1995 to 12/2011 (month/year). Patient clinical and follow-up data were abstracted from both medical records and an institution based tumor registry that retrospectively maintains clinical outcomes. The diverticula and/or intradiverticular tumors were all clinically diagnosed by preoperative cystogram, cystoscopy, computerized tomography scan or magnetic resonance imaging. Tumor grading and staging were based on current World Health Organization (WHO) classification and AJCC manual (7^th^ edition). Surgical procedures performed included 6 radical cystectomies (27%), 7 diverticulectomies/partial cystectomies (32%) and 9 transurethral resection (TUR) specimens (41%). Hematoxylin-eosin slides were re-reviewed by two genitourinary specialized pathologists (HZ and BX) for all 22 cases. In cases with multifocal intradiverticular lesions, the one with higher grade and higher stage was used in further analysis. For histomorphological comparison purpose, four benign bladder diverticular cases and 8 cases of radical cystectomies treated for conventional urothelial carcinoma (not associated with diverticulum) were randomly selected during the same period of time.

### Statistical methods

Continuous variables were reported as mean value +/− standard deviation (SD) and/or median value. The Kaplan-Meier method was used to estimate survival from the date of therapeutic procedure to the end point in the entire cohort and in the subsets of patients. All analyses were conducted in the MedCalc statistical software with a nominal significance level of 0.05. Date of death due to bladder cancer was used as the endpoint in estimating the overall survival. The endpoint in estimating disease free survival was disease recurrence including positive malignant cells in urine cytology specimens or disease progression (metastasis with or without evidence of local disease recurrence). The local disease recurrence included all lesions identified in the remaining urinary bladder after surgical procedures and adjuvant therapies.

## Results

### Clinical features

All patients were Caucasian and all but one were male. Patient age ranged from 39 to 85 years, with a mean of 68 years (median 70 years). Tumor sizes ranged from 0.5 to 5.5 cm, with a mean of 2.2 cm (median 1.9 cm). Locations of the intradiverticular lesions were specified in 20/22 cases (91%). The majority of tumors (15/20, 75%) involved the lateral walls, including the left lateral (7/20), right lateral (7/20), or bilateral (1/20). Less common locations involved the right base (1/20), right anterior (1/20), posterior (1/20), dome (1/20) or trigone (1/20). The number of lesions was specified in 18/22 cases (82%). The majority of tumors (14/18, 78%) were solitary within a single diverticulum (Table 1). Multifocal intradiverticular lesions were grossly identified in four cases. One case had two diverticula, with each diverticulum involved by a solitary tumor.

**Table 1:** **Clinical and histological features of intradiverticular urothelial carcinomas**

**Clinical and Histological Features**	**Cases**
**Total**	**22**
Sex (No.)/Age, mean (range)	68 (39–85)
Male (n = 21)	
Female (n = 1)	
Race, No. (%)	
Caucasian	22 (100%)
Location of the lesions, specified, No. (%)	20 (91%)
Lateral	15 (75%)
Left lateral	5
Right lateral	4
Bilateral	1
Left anterolateral	1
Left posterolateral	1
Right posterolateral	3
Right anterior	1
Posterior	1
Right base	1
Dome	1
Trigone	1
Diagnosis, No. (%)/Specimen type, No.	
Non-invasive papillary urothelial carcinoma	9 (41%)
TUR only	7
Diverticulectomy	2
Infiltrating urothelial carcinoma	10 (45%)
TUR only	1
Partial cystectomy	3
Radical cystectomy	6
Small cell carcinoma	3 (14%)
TUR only	1
Diverticulectomy	1
Partial cystectomy	1
Pathological stage*, No. (%)	
pTa	9 (41%)@
pT1	8 (36%)
pT3	5 (23%)

No. or n indicates the number of cases.

TUR indicates transurethral resection of bladder tumour.

*The tumor staging is based on the current World Health Organization (WHO) classification/AJCC manual 7^th^ edition.

@ 9 cases of pTa (based on the intradiverticular pathological staging), 2 out of 9 these cases were pT1 in their extradiverticular lesions. Survival statistics was based on the higher staging regardless of the tumor locations.

### Histologic features

**Figure 1:**
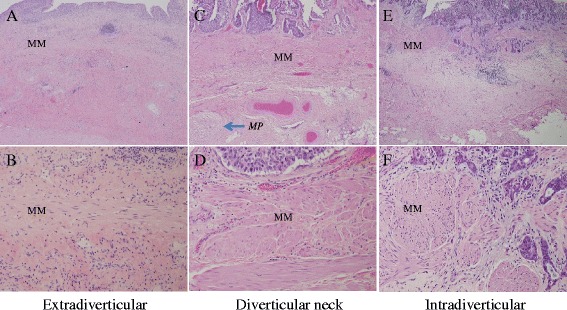
**Representative sections from a radical cystectomy specimen with intradiverticular urothelial carcinoma.** The H&E slides are from extradiverticular **(A and B)**, diverticular neck **(C and D)** or intradiverticular **(E & F)** regions with low (**A**, **C**, **E** – 40X) or higher magnifications (**B**, **D**, **F** – 200X). MM: Muscularis mucosa; MP: Muscularis propria.

Histologically, 9 cases (41%) were non-invasive papillary urothelial carcinomas, 10 cases (45%) were invasive urothelial carcinomas and 3 cases (14%) were invasive small cell carcinomas. All invasive tumors and 6/9 (67%) non-invasive ones were high grade carcinomas. Six out of 10 invasive urothelial carcinomas had coexisting minor histological growth patterns (2 micropapillary, 2 non-invasive papillary, 1 glandular plus chondroid and 1 glandular plus tubular). Two cases of small cell carcinoma had coexisting high grade invasive urothelial carcinoma; one with additional glandular differentiation. Urothelial carcinoma in situ (CIS) was present in 3/10 (30%) invasive urothelial carcinoma and all 3 small cell carcinoma cases. Of 13 invasive carcinomas, including either urothelial carcinoma or small cell carcinoma, 8 cases were T1 and 5 cases were T3 tumors (Table 1). Synchronous extradiverticular urothelial carcinomas were observed in 6 out of 9 cases (67%) of non-invasive and 2 out of 10 cases (20%) invasive intradiverticular urothelial carcinoma, respectively. More than half of the cases (13/22, 59%) showed a distinctive hypertrophic muscularis mucosae. Among them, 8 cases were invasive urothelial carcinomas, 4 were non-invasive urothelial carcinomas, and 1 was the small cell carcinoma with coexisting high grade urothelial carcinoma component. In contrast to normal appearance of muscularis mucosae which was often found in loose subepithelial connective tissue (lamina propria) and retained some distance from the overlying urothelium, the hypertrophic muscularis mucosae was usually located immediately beneath the urothelial mucosae and often above vasculatures in the lamina propria (Figure 1). The hypertrophic muscularis mucosae was mostly disorganized (multiple polarities) in a continuous pattern, as oppose to normal muscularis mucosae that is often organized (single polarity) with discontinuous pattern. In partial and radical cystectomy specimens, the hypertrophic muscularis mucosae were frequently seen starting from the intradiverticular neck region. In addition, prominent vessels or even hypertrophic vessel walls were often present within the diverticula. The bundles of hypertrophic muscularis mucosae were sometimes randomly mixed with the infiltrating tumor cells. Occasionally, isolated small bundles of muscularis propria were found in the diverticular neck region.

**Figure 2:**
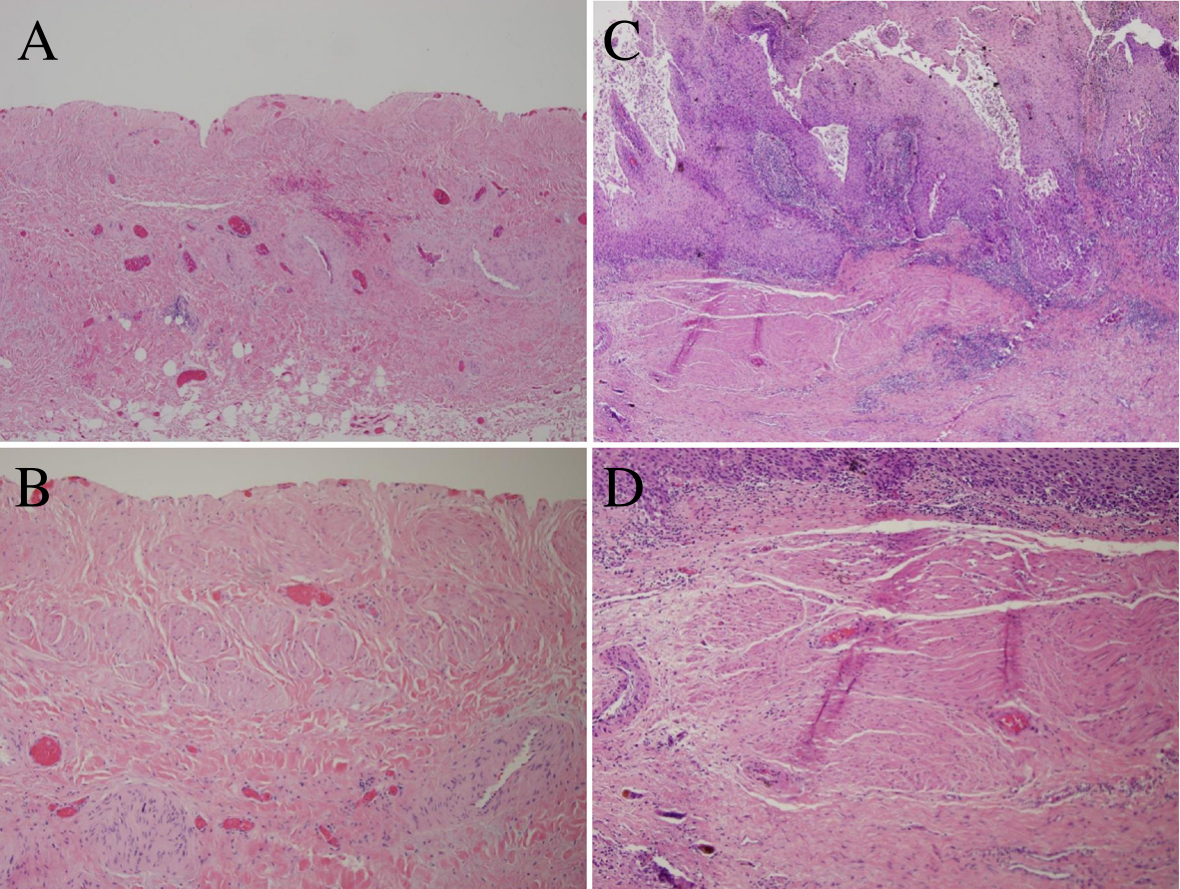
**Representative sections of hypertrophic muscularis mucosae.** The H&E sections from bladder wall of a benign bladder diverticulum with completely denuded urothelium (upper tissue edge), showing hypertrophic muscularis mucosae **(A and B)**. **C** and **D** are from the conventional (lesion not arising from diverticula) neoplastic bladder wall of a well-differentiated squamous cell carcinoma, showing hypertrophic muscularis mucosae. Low (**A**, **C** – 40X) or higher magnifications (**B**, **D** – 100X), respectively.

Hypertrophic muscularis mucosae are not unique to intradiverticular malignancy as it can be also found in bladder diverticula with benign conditions or prostatic disease. Of randomly selected 4 benign bladder diverticula and 8 radical cystectomy **S**pecimens without diverticulum (Figure 2), hypertrophic muscularis mucosae was identified in one out of 4 benign bladder diverticula and 2 out of 8 radical cystectomy specimens treated for conventional (not associated with diverticulum) urinary bladder carcinoma.

### Clinical follow-up

Clinical follow-up was available for all 22 patients, with a mean follow-up of 38 months (range 1–135 months, median 32 months). Of the 9 patients with non-invasive papillary urothelial carcinoma, two were free of disease at last follow up; two died of other disease with no evidence of recurrence; five developed recurrences within the bladder. Recurrent disease was treated with transurethral resection (TUR) (N = 3) or diverticulectomy (N = 2). Among 3 TUR treated patients, two died of disease with one experienced grade progression and developed metastases to the lung and liver, and the other one developed lymph node metastasis. Of two patients treated with diverticulectomy, one recurred after 33 months and the other one remained disease-free after 40 months.

In 10 patients with infiltrating urothelial carcinoma, all but one were treated with partial (N = 3) or radical cystectomy (N = 6), while the remaining patient underwent conservative TUR management. Half of these 10 patients were free of disease till the last follow-up which was a median of 51 months; three died of disease at 17, 26 and 41 months after surgery with distant metastases; one recurred with local disease after 18 months but was alive 20 months after surgery; one developed lung metastasis after one month but was alive 10 months after surgery. Among 3 patients with small cell carcinoma, two of them recurred shortly after surgical procedures at 1 and 4 months. One died of disease with disseminated metastases 13 months after surgery.

**Figure 3:**
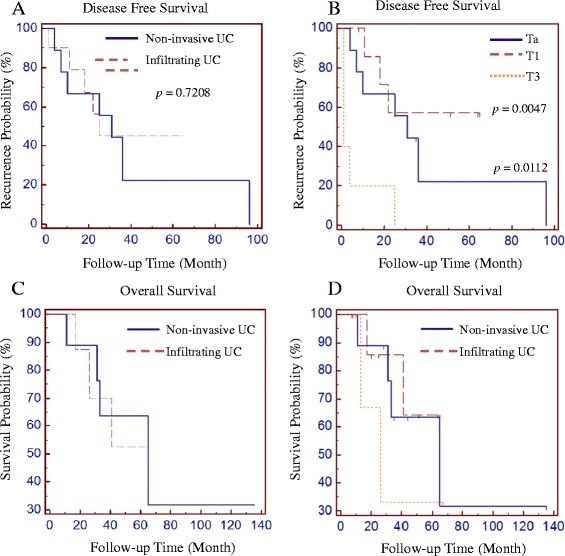
**Comparison of disease free survival (A and B) and overall survival (C and D) among patients with different histological types (A and C) or with different pathologic stages (B and D) of intradiverticular bladder carcinomas.** In Figure 3**B**, Recurrence probability was compared between T3 and Ta (*p* = 0.0112), or T1 (*p* = 0047). Recurrence probability was not statistically different between Ta and T1 (*p* = 0.4156).

Disease free survival time was similar between patients with non-invasive and infiltrating urothelial carcinoma (median disease free survival time 31 versus 29 months, *p* = 0.7208) (Figure 3A) or between patients with Ta and T1 tumors (median disease free survival time 31 versus 24 months, *p* = 0.4156) (Figure 3B), but significantly different among patients with pT3 tumour (median disease free survival time 1 month) compared to those with pTa (*p* = 0.0112, Hazard ratio 3.3897, 95% CI 0.7679 – 14.9638) or pT1 (*p* = 0.0047, Hazard ratio 4.9579, 95% CI 0.9721 – 25.2819) (Figure 3B). The differences of overall survival time, however, were not statistically significant between non-invasive and invasive carcinoma, or among different subgroups of carcinoma within the follow-up period (Figure 3C and D).

## Discussion

The earliest literature describing a diverticulum of the urinary bladder dates back to a century ago [1,9], documenting discovery of the unique anatomic and histological features of the congenital (muscularis propria present) or acquired (muscularis propria absent) diverticulum. There has since been little information, however, on the surgical management, pathological staging and prognostic features of bladder carcinoma arising in this unusual setting due to scarcity of sufficient cases and focused investigation. Our study supports findings that lamina propria layer within the diverticulum commonly features hypertrophic muscularis mucosae, with the normally thin and wispy smooth muscle fiber layer taking on an unusually haphazard arrangement and irregular shape [4,10-12].

Accurate recognition of hypertrophic muscularis mucosae has key clinical implications. Since the hypertrophic muscularis mucosae may morphologically resemble muscularis propria, it is not uncommon to lead to a misinterpretation of pathologic staging of the tumour particularly in TUR specimens, resulting in inappropriate staging [13]. To date, there are no clear histological criteria to define the hypertrophic muscularis mucosae. In recent years, extensive efforts have aimed to employ smoothelin as an immunohistochemical marker to differentiate muscularis propria from the muscularis mucosae. Although smoothelin stain appears to be somewhat useful to distinguish muscularis mucosa from muscularis propria, the intensity of smoothelin expression in the muscularis propria appears to be similar as compared to that in the hypertrophic muscularis mucosae. It is reported that hypertrophic muscularis mucosae show 2+ smoothelin staining in one third of the specimens tested [11]. Therefore, it is our opinion that careful histomorphologic examination is still the most reliable method to distinguish hypertrophic muscularis mucosae from muscularis propria.

In our current study, all specimens with full thickness bladder wall resection did not show definite intact muscularis propria within diverticula, presumably reflecting acquired rather than congenital diverticula. A few radical cystectomy specimens showed irregular layers of muscularis propria adjacent to the diverticular neck region (Figure 1C). In contrast, hypertrophic muscularis mucosae was identified in more than half cases (13/22, 59%). We summarize the following histological features to aid in identification of hypertrophic muscularis mucosae: 1) hypertrophic muscularis mucosae is usually located immediately beneath the urothelial mucosae above the lamina propria vasculature; 2) the hypertrophic muscularis mucosae is usually disorganized (multiple polarities or non-linear) but shows more continuous pattern compared to the normal muscularis mucosae; and 3) the muscularis mucosae often becomes hypertrophic starting from the intradiverticular neck region.

The cause of hypertrophic mucosae is most likely multifactorial. Weakened urinary bladder wall in diverticulum due to lack of the muscularis propria is likely the main mechanism that causes hypertrophic muscularis mucosae, which can therefore compensate for the lost strength of the bladder wall in a diverticulum. In addition, the contraction of bladder will cause stretching of the muscularis mucosae in the diverticulum and Frank-Starling’ law may come into play for output of urine from this region. Ultimately, this area develops hypertrophied muscularis mucosae over time. The prominent vessels and/or hypertrophic vessel walls within the diverticula as demonstrated in current study may be additional compensatory mechanisms involved. Other mechanisms underlying hypertrophic muscularis mucosae may include urinary outflow obstruction, local chronic irritation and others. At molecular level, growth factors have been demonstrated to be able to stimulate muscle particularly smooth muscle proliferation predominately through activation of mitogen-associated protein kinases [14-16]. These growth factors, especially TGF- β, are frequently shown to be expressed at high levels in and promote the progression of urothelial carcinoma [17]. Therefore, it is reasonable to speculate that these growth factors can be synchronously involved in hypertrophic process of the muscularis mucosae. In our study, cases with hypertrophic muscularis mucosae are found in 8 out of 10 infiltrating urothelial carcinomas (80%), 4 out of 9 non-invasive carcinomas (44%) and 1 out of 3 small cell carcinomas (33%). Although the hypertrophic muscularis mucosae is not specific to any particular pathophysiolocal conditions, one should realize that it is very likely present in intradiverticular invasive urothelial carcinoma specimens, with much higher frequency than other bladder lesions - either benign or malignant. Recognizing this histological landmark is critical in surgical pathology practice, particularly in the setting of intradiverticular urothelial carcinoma.

Based on current practice, intradiverticular urothelial carcinoma is often staged and treated aggressively as it is thought to be more likely to disseminate [8,18,19]. However, clinical outcome in patients with carcinoma arising in bladder diverticula is uncertain due to insufficient case number and short follow-up time in the published literature. The current study was limited to predominantly Ta and T1 patients, and follow-up data was available on all 22 patients with on average 3 years. Given most recurrences occur within one year of various treatment modalities, this follow up length allows for certain inferences to be made. Total 10 patients were classified as T1 in clinical follow-up, including 8 with T1 tumour within diverticula and 2 with Ta tumour within diverticula but T1 extradiverticular lesion. Total 9 patients were classified having Ta tumors. Our current study shows that patients with Ta and T1 tumors share similar chance for disease recurrence, which is probably due to that those T1 patients benefit from more aggressive managements including radical cystectomy performed in half of these patients (Table 1). However, patients with T3 disease in this study had poor disease free outcomes in comparison with those with T1 disease as expected, despite common bladder-sparing approaches as definitive treatment for both groups. While our conclusions are limited by sample size, follow up interval, and absence of T2 disease for comparison, these findings suggest the missing muscularis propria layer may not necessarily confer more disease recurrence behavior to T1 lesions. Whether the hypertrophic muscularis mucosae play a role in bladder cancer with local invasion remains to be investigated. Furthermore, some of our cases actually had multiple resections because of recurrence or had additional postsurgical managements, such as BCG therapy. It is not feasible to further subgroup these cases again based on small number of cases. Collaborative multicentric and case–control studies/meta-analyses are now needed to improve understanding of the histopathologic features, biological significances and clinical outcome of intradiverticular urothelial carcinoma in comparison with that of conventional urothelial carcinoma.

## Conclusion

In this retrospective study, we confirmed previous observation that intradiverticular carcinomas are often associated with a hypertrophic layer of muscularis mucosae that can potentially confound tumor staging. Non-invasive intradiverticular urothelial carcinomas are more likely to have coexisting synchronous extradiverticular lesions. The absence of a muscularis propria layer may not necessarily predispose T1 tumors to more aggressive disease.
